# Transactions of the British Medical Association

**Published:** 1895-11

**Authors:** 


### Transactions of the British Medical Association.

[From advance sheets sent us by the author.]

### ELECTROTHERAPY AS A MEANS OF DIAGNOSIS IN GYNECOLOGY.

*By Dr. G. Apostoli, of Paris.*

Dr. Apostoli, after a long and thorough trial of his method, has come to the following general conclusions:

1. The faradic current of tension (generated by the coil of long and fine wire) applied to the uterine cavity, according to the rules established by Dr. Apostoli in 1883, relieves, for a longer or shorter time, all ovarian pain of nervous or hysterical origin; but remains powerless or nearly so in cases of ovarian pain caused by inflammatory lesion of the peri-uterine tissue or of the appendages.

2. The same faradic current is therefore useful in diagnosis, inasmuch as it helps us to distinguish the nature of so-called ovarian pain, and to determine rapidly the differential diagnosis between hysterical and inflammatory ovarian pain. Where the two kinds of pain exist in the same patient we are helped to understand their nature by the fact the one is relieved and the other is not.

3. If, then, the curative effect of the faradic current clears up or rectifies a doubtful diagnosis, it protects us at the same time, from undertaking a useless operation.

On the other hand, if the same faradic current proves ineffective, the lesion being inflammatory, we are led to resort to a supplementary galvanic treatment or to a surgical operation sooner or later.

4. The constant galvanic current, applied to the uterine cavity in doses gradually increasing from 50 to 120 milliamperes, according to the rules published by Dr. Apostoli in 1884, and bearing in mind the individual susceptibility and tolerance, will be almost always supported without much pain during the seance, and without febrile reaction afterward, if the parts adjacent to the uterus are free from inflammation.

Simple cystic, peri-uterine tumors, which are neither inflamed nor suppurating (such as ovarian cysts and hydro-salpinx); may also show perfect tolerance of the galvanic current.

The galvanic-current is also sometimes perfectly supported by cases in which the uterus is surrounded by old inflammatory products or exudations no longer pathogenic.

5. There are three classes which should be considered as exceptions to the preceding rule, for they bear the galvanic current more or less badly, though they do not necessarily produce much febrile reaction after the seance.

They are:

A.—Certain forms of hysteria.

B.—Fibro-cystic tumors of the uterus.

C.—Enteritis with false membrane.

It is generally easy to diagnose these cases of intolerance.

6. All acute peri-uterine inflammation (of the pelvic cellular tissues, of the peritoneum and especially of the appendages) will cause the galvanic current to be badly borne when it passes 40 or 50 milliamperes, and will cause intolerable pain and febrile reaction when carried beyond this intensity.

7. The intolerance for the galvanic current is generally proportionate to the extent and gravity of the lesions referred to and increases with the intensity of the current employed—especially when it passes 40 or 50 milliamperes.

8. All inflammation of the appendages which is *curable* (*symp-*

*tomatically at least*) without radical operation, will bear the galvanic current better and better, and there will be a corresponding improvement of the prominent symptoms such as pain and hemorrhages.

The intolarance noted at the beginning progressively disappears.

9. All grave inflammatory lesions of the appendages, and notably all suppurative processes which are *incurable* (*even symptomatically*) by conservative means, show the same intolerance from the beginning to the end of the treatment which was noticed at first, and which is apt to increase instead of diminish if the treatment is continued.

10. Thus, the simple study of the tolerance or intolerance of the intra-uterine galvanic treatment, and especially of the post-operative pain and fever occurring on the evening of, or the day following the treatment, enables us to make the diagnosis. It also, in 4 or 5 seances, given twice weekly, informs us of the condition of the appendage, of their possible inflammation and its degree, and in this way it lessens the number of laparatomies and exploratory incisions.

11. The same study of the so-called galvanic reactions also informs us rapidly (in 5 to 10 seances) of the curability of these inflammatory lesions which the electric current has demonstrated, and in consequence of this it tells us in one case to abstain from operation while in another it shows an operation to be urgent.

En résumé, gynecological electro-therapeutics, carefully methodically and patiently applied, instead of being opposed to the marvellous progress of surgery, comes to its aid.

Independently, in fact, of the great therapeutic service which it renders every day, electricity serves as a *touch-stone*; it assists us in diagnosis and thus directly serves the interests of surgery, in one case showing an operation to be useless and dangerous, in another that its necessity is urgent.

Thus many of laparotomies, so-called exploratory incisions ane mutilations practiced without due deliberation for the relief of rebellious ovarian pain or for lesions of the appendages of uncertain nature, should be, from this time forth, delayed or formally proscribed until all the resources of *faradic sedation* on the one hand, and of the *intra-uterine galvanic effect* on the other, have been tried. Experience has abundantly proved these currents to be innocuous, if given with necessary aseptic precautions.

### THE GENERAL THERAPEUTIC EFFECT OF THE ALTERNATIVE ELECTRIC CURRENT OF HIGH FREQUENCY AND OF HIGH TENSION.

Dr. Apostoli, together with Dr. Berlioz, on the 18th of March, 1895, presented a paper on the above mentioned subject to the Academy of Sciences of Paris. He now, after longer and riper experience, desires a summary statement of his general conclusions:

1. According to Professor d'Arsonval's discoveries alternative currents of high frequency and of high tension, exert a powerful action upon all living bodies submitted to their inductive influence.

2. The best method of applying these induced currents is to place the patient, free from all contact with electrodes, in the circuit of a large solenoid traversed by these currents.

The patient being thus completely insulated, the currents, which circulate in his body by *auto-conduction*, have their origin in his tissue. The body plays the role of a closed induced circuit.

3. By this method the physiological discoveries of Professor d'Arsonval are confirmed, and we are able to prove the powerful influence of these currents upon the *vaso-motor* system—although they produce absolutely no sensation and although they have no apparent effect upon the motor or sensory nerves.

These currents have nevertheless a powerful action upon all the nutritive functions; as has been verified by Dr. d'Arsonval's numerous analyses of the gaseous products of respiration and by Dr. Berlioz's not less numerous analyses of the urinary excreta.

4. The general therapeutic applications to be deduced from this physiological action are confirmed by clinical observation.

Dr. Apostoli has now treated more than a hundred patients by this method at his clinic and at his private consultation rooms. The greater number of these patients have been greatly benefited by this new treatment, which, be it remarked, has been used to the exclusion of all other forms of medication, dietetic or otherwise.

5. These currents exert in the majority of cases a most powerful and beneficial action upon diseases due to *slackening of the nutrition* by accelerating organic exchanges and combustion. This is proved by analyses of the urine made by Dr. Berlioz, of which the following is a brief resumé:

The quantity becomes more normal; the products of organic waste are better eliminated.

The *increased combustion* is shown by the diminution of *uric acid*, while the percentage of *urea* is generally increased. The relative proportion of these two substances changes under treatment so as to reach in general the figure of 1.40.

The elimination of the mineral products is also changed, but in a manner less marked.

6. When daily séances are given, each lasting fifteen minutes, we may generally observe in patients, submitted to the influence of these currents, the following modifications in their general condition. We mention them in the order of their occurrence:

Return of sleep.

Increase of strength and vital energy.

Increase of gaiety, of power for work and ability to walk.

Improvement of appetite, etc.

In short, *general progressive improvement.*

This general improvement often manifests itself after the first seances, before any local influence is apparent and before any change has occurred in the urinary secretions.

7. Local pain and trophic changes are often more slowly affected by these currents, and at times they are entirely refractory for a longer or shorter period.

In such cases the same currents must be applied locally by contact with the electrodes.

This subject will be treated later on in a separate communication.

8. The diseases which have appeared incurable by this treatment are those not associated with well defined organic changes, such as *hyteria* and certain forms of *neurasthenia.*

Dr. Apostoli has also observed that certain *localized neuralgias* are refractory to this form of currents, they require its more direct local application.

9. The diseases which have derived most benefit from this therapeutic agent, belong to the *arthritic class: rheumatism* and *gout.*

10. In certain *diabetic* subjects the sugar has disappeared altogether from the urine under the influence of these currents, while in others there has been no such change, nothwithstanding the manifest and constant improvement in the general condition.

Is this difference due to the imperfection of the electric apparatus or to the manner of its application? It is hoped that further experience will soon afford an answer to this question; although the fact that diabetes has many different causes, may in itself explain the difference in the results obtained by this treatment.

11. In conclusion, the currents of high frequency and of high tension introduced into electro-therapeutics by Dr. d'Arsonval greatly increase the field of action of medical electricity.

They furnish general medicine with a new and valuable means of treatment, capable of modifying more or less profoundly the processes of nutrition.

